# Link Prediction Between Structured Geopolitical Events: Models and Experiments

**DOI:** 10.3389/fdata.2021.779792

**Published:** 2021-11-30

**Authors:** Mayank Kejriwal

**Affiliations:** Viterbi School of Engineering, Information Sciences Institute, University of Southern California, Los Angeles, CA, United States

**Keywords:** event representations, representation learning, geopolitical event link prediction, word embeddings, multi-partite networks

## Abstract

Often thought of as higher-order entities, events have recently become important subjects of research in the computational sciences, including within complex systems and natural language processing (NLP). One such application is event link prediction. Given an input event, event link prediction is the problem of retrieving a *relevant* set of events, similar to the problem of retrieving relevant documents on the Web in response to keyword queries. Since geopolitical events have complex semantics, it is an open question as to how to best model and represent events within the framework of event link prediction. In this paper, we formalize the problem and discuss how established representation learning algorithms from the machine learning community could potentially be applied to it. We then conduct a detailed empirical study on the Global Terrorism Database (GTD) using a set of metrics inspired by the information retrieval community. Our results show that, while there is considerable signal in both network-theoretic and text-centric models of the problem, classic text-only models such as bag-of-words prove surprisingly difficult to outperform. Our results establish both a baseline for event link prediction on GTD, and currently outstanding challenges for the research community to tackle in this space.

## 1 Introduction

**FIGURE 1:**
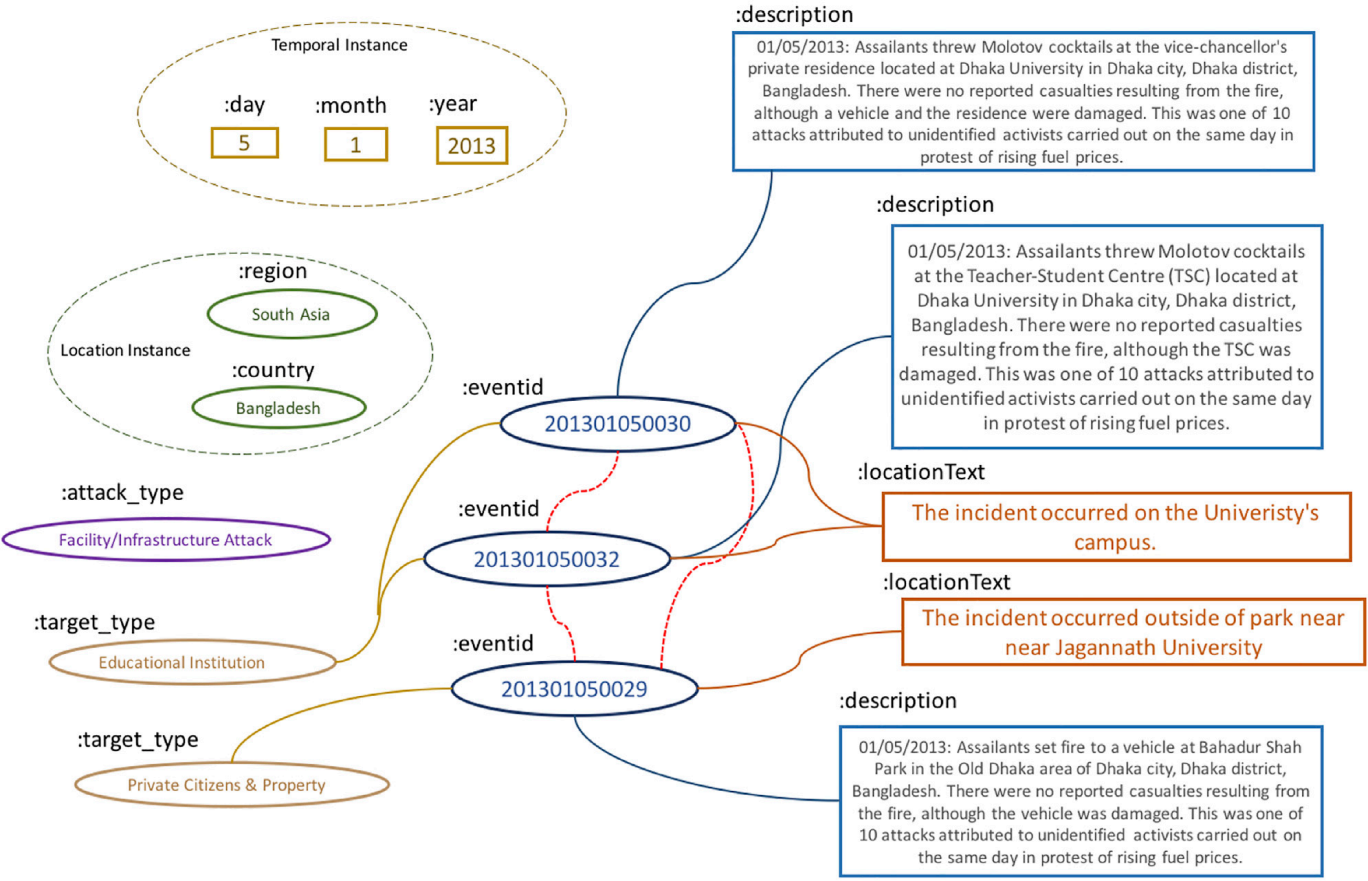
Illustrations of three geopolitical event fragments that are linked.

In recent years, there has been an increasing focus on representing, reasoning over and doing inference on, *events* Lorenzini et al. (2021), Battistelli et al. (2013). Unlike ordinary named entities, events are complex data structures, embodied by artifacts such as triggers, actors, locations, descriptions, and spatiotemporal cues. In the case of events with geopolitical consequences, such as terrorist attacks, assassinations, or bombings, automatically and accurately predicting links between events is an important research application that can be used to populate and enrich geopolitical, sparse (and proprietarily gathered) knowledge bases with global scope. Figure 1 provides a simplified illustration, based on real data, of a linked set of events.

For such inferential tasks, Representation Learning (RL), an important sub-area of deep learning research, has emerged as extremely influential in both graph- and text-centric communities. In the general case, latent space embeddings (dense, real-valued vectors) are learned on graphs with simple structures, or [in the case of text embedding algorithms like GloVe and word2vec Pennington et al. (2014); Mikolov et al. (2013)] on word or character sequences. On the other hand, structured event data contains rich structure and semantics that can be exploited to learn better representations.

As one important event-centric application, consider *geopolitical forecasting*, which continues to be an important and relevant problem, especially for policy institutes, think tanks, analysts and pollsters Tetlock (2017), Tetlock (1992). The problem is also known to be difficult, although the geographic region and complexity involved in making a forecast for an IFP (individual forecasting problem) can make some forecasts more challenging than others Tetlock (2014). One of the critical tasks of forecasting is to distinguish relevant material from the irrelevant. This is especially true at the level of events e.g., when one is trying to forecast political instability in Nigeria, it is helpful to consider “prototypical political instability” events like riots or protests, and retrieve both recent such events, as well as links to other events that have a connection to the prototypical event Esser and Strömbäck (2013), Zinken (2003).

Obtaining and reasoning over such contextual and background knowledge is ever more important also because (arguably) it is becoming harder to make accurate forecasts, even for events that are being globally scrutinized and studied. In 2016, several incidents occurred globally that went against the predictions of famous (i.e., traditionally accurate) polls^1^, including the outcomes of Brexit and the 2016 US Presidential Election. While these examples may arguably be described as representing extremes [“Black Swan” incidents Nassim (2007)], *consistently* making correct forecasts is a valued skill that several individuals (and by extension, organizations) have been known to possess Tetlock and Gardner (2016). A key differentiator between successful (on average) forecasters and ordinary forecasters is the ability to consider contextual and linked information when researching the forecasting problem. Events, such as COVID-19 and the US Capitol Hill riots following the Presidential 2020 election, only serve to highlight the severity and suddenness of Black Swan events.

We also note that while, on the surface, tasks like event link prediction may sound similar to link prediction as studied in social networks, the complex structure of an event makes the former much more difficult and ill-defined. This is also true for other event-centric problems like event resolution Kejriwal et al. (2018b), event extraction (in the natural language processing community) and event co-referencing Ng (2017), Lu and Ng (2017), for which special techniques have now been developed, as well as situational awareness and visualization Kejriwal et al. (2018a), Kejriwal and Zhou (2019).

In this paper, we address the research problem of what features make for good event representations, both when text summaries are available, or unavailable. We propose and consider several models of events, including models that just use a short text description, a combination of text and locations, paths in a multi-layer semantic network, or in the most general case, novel models that rely on various statistical-semantic cues in both text- and graph-theoretic frameworks. Using both classic methods, such as cosine similarity applied on bag-of-words vectors, as well as deep embedding methods, we study and contrast these representations by conducting a full set of event link prediction experiments on the Global Terrorism Database (GTD) LaFree and Dugan (2007). Our goal here is not to present novel algorithms but to introduce and present a rigorous methodology (including data and evaluation metrics) for studying event link prediction as a fundamental application area in multi-relational networks and complex systems.

Using various metrics inspired by the information retrieval and traditional link prediction communities Liben-Nowell and Kleinberg (2007), we quantify the most salient aspects in learning good event representations, especially when a combination of structured and unstructured information sets may be available. To the best of our knowledge, this is the first such study to rigorously model, formalize and quantify event representation learning.

## 2 Problem Description

We begin by first defining and scoping the notion of an *event* as assumed by this article. If events were completely arbitrary, it would be sufficient to assume an event ontology (EO), and declare instances defined in terms of certain classes (event types and sub-types) in the EO to be “events”. In the real world, however, events, despite exhibiting heterogeneity across sources, domains and datasets, do obey some near-universal restrictions. First, events are generally *typed*, whether automatically or not. For example, the Global Terrorism Database (GTD) LaFree and Dugan (2007) describes terrorism events, as the name suggests, while datasets like the Armed Conflict Location and Event Data Project (ACLED)^2^ or Political Instability Task Force (PITF)^3^ contain a mix of events that are useful to geopolitical analysts. A good example of an ontology describing many event types, and that has been extensively used in the real world, is Conflict and Mediation Event Observations (CAMEO)^4^.

Second, a commonality between databases that describe geopolitical events is some notion of space and time. Although the granularity can differ (e.g., some highly proprietary event datasets may be associated with very precise geo-coordinates, while many others contain coarser information, like region and country names), some spatial and temporal information is almost always included. Third, depending on the event type, some structured information could be encoded using a highly controlled vocabulary. In ACLED, very specific (and consistent) terminology is used to indicate event modalities like riots or protests, for example, while in GTD (as we later describe), information like the attack type and target type obey a controlled vocabulary that is given by a codebook.

At the same time, events that have different modalities or provenance can also be very heterogeneous. Thus, it is important to be flexible in an event formalism to accommodate the ‘quirks’ of individual datasets. With this caveat in mind, we can use the three observations above to define a geopolitical event *E* in the following way. Given an event ontology *O*, a geolocation ontology *G* and a temporal ontology *T*, a *geopolitical event* *E* may be defined as an *instance* of *O* with a spatiotemporal span <g,t> , with *g* and *t* being instances of *G* and *T* respectively. A good example of *G* is the GeoNames ontology Wick (2006), which is widely used in spatial sciences and geography. However, *G* can also be an ontology that is extremely fine-grained such as the underlying ontology behind systems like Google Maps and OpenStreetMap (OSM) Haklay and Weber (2008). In contrast, temporal ontologies are usually simple, although sophisticated options have been proposed in the literature Hobbs and Pan (2006).

We note that, while this description abstracts the full scope of event databases (which can contain tens, if not hundreds, of fields in their schemas), it does not abstract away the fact that events are complex data structures that can contain a combination of free text, structured elements (such as date and location) and elements from controlled vocabularies (such as attack types for terrorist events). Unlike natural language text, or RDF graphs, it is not clear how to *model*, and *learn representations* for, events in a way that makes them amenable to advanced machine learning-centric analytics like link prediction, event classification or anomaly detection^5^. In keeping with established terminology, the learned representation of an event intuitively corresponds to a “feature vector” that can be used in (either supervised or unsupervised) machine learning systems for various classification and clustering problems.

With the advent of deep learning and embedding methods, modeling and representation have become linked. Intuitively, modeling an event (for the purposes described above) defines which *information sets* of an event are relevant, and what the relationships are between these information sets. We consider some models and information sets in a subsequent section. The representation learning is the application of an algorithm (whether developed from scratch, or from the literature, like word2vec) on a modeled set of events. In Natural Language Processing (NLP) terminology, modeling determines the definition of a context^6^, while representation learning uses the context to embed events into a vector space, which is usually (but not always, as we explain later) dense and real-valued.

## 3 Materials and Methods

### 3.1 Global Terrorism Database (GTD)

Before describing the models, we start by describing an important dataset called Global Terrorism Database (GTD) that contains thousands of structured terrorism events spanning the globe, typed according to an expansive schema. As described on the project page^7^, GTD is an “open-source database including information on terrorist events around the world from 1970 through 2016 (with annual updates planned for the future). Unlike many other event databases, the GTD includes systematic data on domestic as well as international terrorist incidents that have occurred during this time period and now includes more than 170,000 cases”.

Because GTD is relatively clean, it allows us to quantify many of the (subsequently discussed) models without being concerned about bias being caused by specific kinds of noise that are usually non-random and caused by imperfect information extraction algorithms (that are still active areas of research). The dataset profile is provided in Table 1, categorized by *attack types* (Table 2). The profile shows that GTD is quite diverse, and spans multiple world regions and countries, a range of target types, and covers events that are fairly recent, allowing us to relevantly extend the conclusions drawn in this article to modern-day events.

**TABLE 1:** A profile of GTD events, categorized by attack types. The number in the first column is an attack type code; see Table 2 for the codebook.

Attack type	Num. events	Num. unique regions	Num. unique countries	Num. unique target types	Date range
0	41	9	21	4	2013/01/21- 2016/12/01
1	3,478	11	79	18	2013/01/01- 2016/12/31
2	12,856	12	98	21	2013/01/01- 2016/12/31
3	29,683	12	101	21	2013/01/01- 2016/12/31
4	144	9	28	15	2013/02/06- 2016/12/23
5	205	10	39	18	2013/01/16- 2016/12/23
6	4,239	10	62	20	2013/01/01- 2016/12/31
7	2,675	12	89	22	2013/01/01- 2016/12/28
8	251	10	41	13	2013/01/08- 2016/12/26
9	2,791	9	55	19	2013/01/10- 2016/12/31

**TABLE 2:** Attack type codebook employed in GTD.

Attack type code	Description
0	Description unavailable
1	Assassination
2	Armed Assault
3	Bombing/Explosion
4	Hijacking
5	Hostage Taking (Barricade Incident)
6	Hostage Taking (Kidnapping)
7	Facility/Infrastructure Attack
8	Unarmed Assault
9	Unknown

As with any study and set of experiments, it is important to keep in mind the limitations of GTD, including data coverage. Currently, it is unknown if GTD is biased toward events of a specific type (whether involving a specific attack type, target type, geographical region, number of individuals involved, and so on), since published research on event databases and their analysis continues to be sparse. We do not claim that GTD is perfect; our goal in using it is to ensure that, caveats notwithstanding, we are able to conduct sufficiently controlled experiments and draw suitable conclusions. Future work will attempt to add more degrees of freedom to these studies.

#### 3.1.1 Link Prediction Ground Truth

**FIGURE 2:**
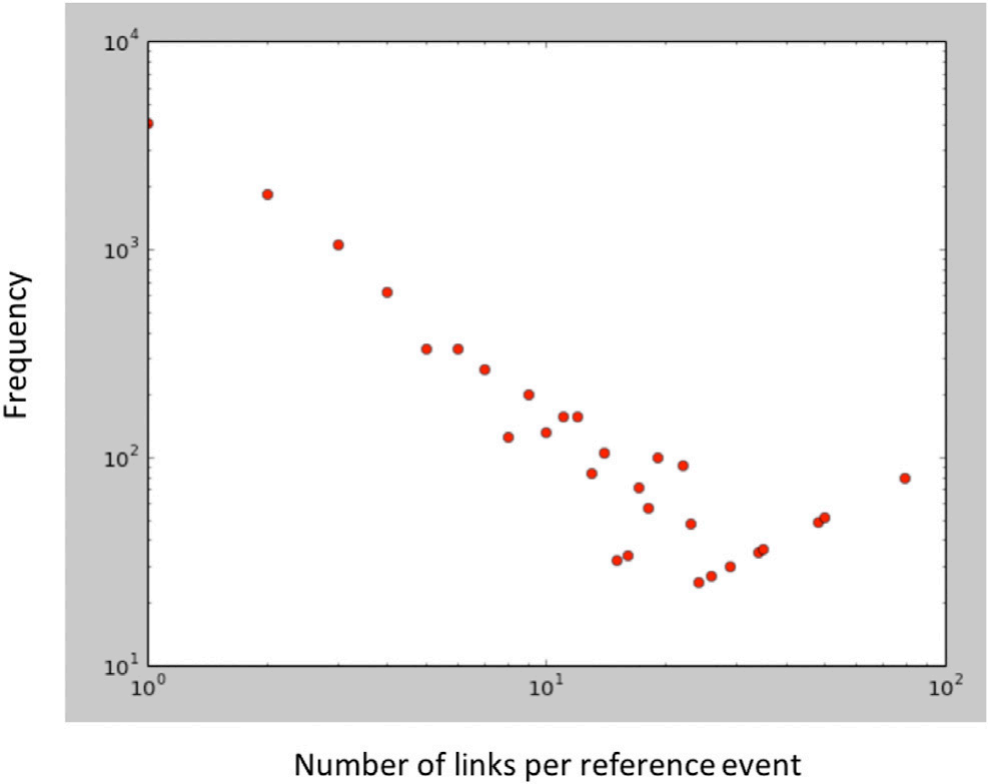
GTD link prediction ground-truth frequency plot.

GTD includes a column that states the event IDs linked with a given event ID. More than one event ID can be linked to a given event ID. In this ground truth, we found that the number of *reference* events^8^ was 10,259 i.e., most of the events in GTD are isolated and are not linked with any other (at least to the extent that it is currently known). The average number of linked events per reference event was found to be 5.204 and the number of *ordered* linked event pairs is 53,392. A frequency distribution is illustrated in Figure 2. By ordered, we mean that a linked pair (*event*_1_, *event*_2_) is considered distinct from (*event*_2_, *event*_1_). There is a reason for this methodology. Usually, in event retrieval scenarios, analysts have an event in mind already and are executing a *more-like-this* task whereby the goal is to retrieve linked events from a database of events. As we describe later, given such a reference event, the correct way to evaluate a retrieval system is to produce a ranked list of candidate events and then quantify the performance of the ranking using information retrieval metrics. Because the rankings are with respect to a reference event, and can be asymmetric (it is possible for *event*_1_ to be ranked high when *event*_2_ is the reference vector, but not the other way around), it is appropriate to consider pairs to be ordered rather than unordered.

### 3.2 Models

Given the event definition in Section 2, we explore in this section the information sets that are expected to be useful for representation learning, especially as applied to a downstream task like link prediction. One reason to consider several possible information sets is that there is a natural tradeoff between including more information in the model, which could lead to richer and finer-grained representations, but that may not generalize as well (especially if some of the information is missing in some records). On the other hand, a model that is too coarse (e.g., that only considers the region in which the event takes place) will likely not be able to distinguish between sufficiently many events and will have poor retrieval performance.

Given that events are usually accompanied by text descriptions in databases such as GTD, the simplest possible information set is *text*. A *text-centric model* can be constructed by simply taking the description (also called the ‘summary’ in GTD) and not assuming or using any other structure.

**FIGURE 3:**
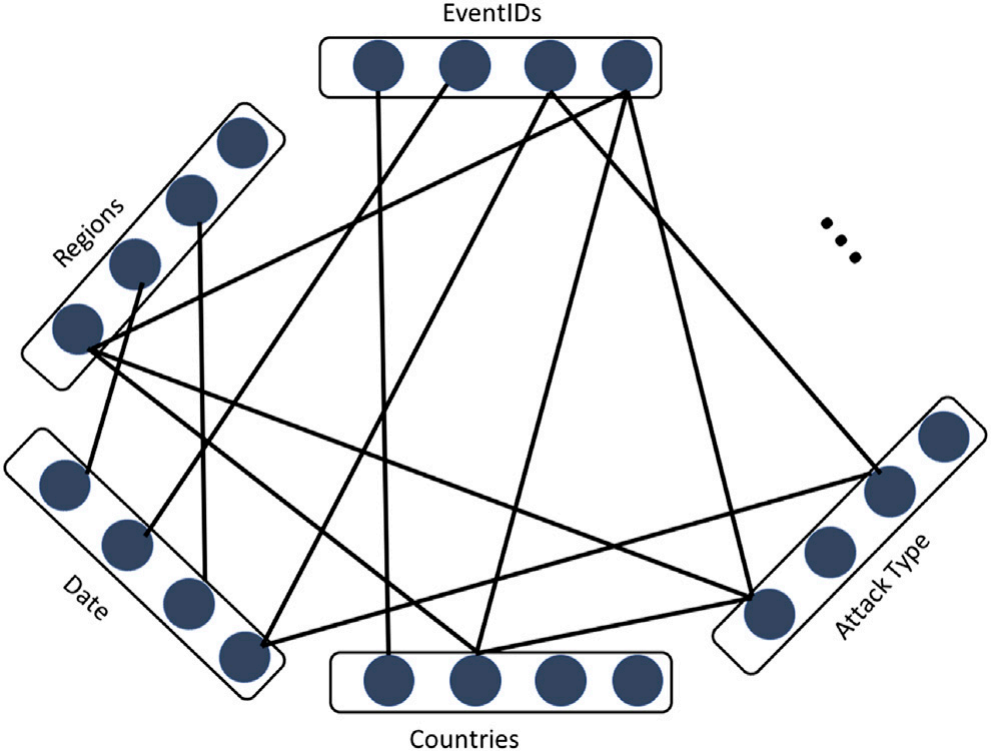
Events modeled as nodes in a multi-partite semantic network (MPSN). We only consider events that have at least one piece of information associated with it (besides an event ID).

At the other extreme is a model that only takes the graph structure into account. We refer to such a model as a *multi-partite semantic network (MPSN)* model, illustrated in Figure 3. Assuming the model is represented as an edge-list, each event is represented using an “event ID” vertex, with edges linking the vertex to any other vertices that characterize the event. An important point to note here is that the different “semantic layers” in the network must form a closed set i.e., the vertices must be pre-specified in advance. This implies that we know the regions, attack types (and so on) that are in our domain. Constructing multi-partite semantic networks over open sets of nodes is not a well-defined problem at the present moment for the purposes of specifying and learning representations on a network.

However, although the text-based and MPSN have their respective advantages, one could presumably aim to take advantage of both. On the one hand, it is difficult to frame the text as a graph without losing its natural language structure, and to frame the graph as text. On the other hand, representation learning (and downstream link prediction) can accept heterogeneous information sets as input. Keeping this in mind, and with a view towards simplicity in the modeling stage, we model an event with both semantic and text information sets as *E* = < *E*_*T*_, *E*_*N*_ >, where *E*_*T*_ and *E*_*N*_ are the text-centric (expressed simply as a string) and MPSN (expressed as a sub-graph of a network such as the one in Figure 3) representations of the event. We refer to each component of the tuple above as an *event facet*.

One can even generalize the notion above, where an event has multiple facets, and each facet captures a unique combination of information sets. For example, we described earlier how multiple text-centric and MPSNs are possible. Rather than pick one or the other, one could consider “bags” of models by including each model as an event facet. Whether it is worthwhile to do so would depend both on how the representation learning processes this data. We provide a brief set of results exploring such “hybrid” models in Section 5.

### 3.3 Representation Learning on Models

Over the last decade, neural network models like skip-gram and continuous bag of words (CBOW) have been used with great effect^9^ to embed sequences and structured data (like graphs) into a dense, real-valued vector space Mikolov et al. (2013). The vectors can be used as features for link prediction. An important question arises as to how to embed each of the event models described in the previous section. For text-only models, an appropriate neural text embedding such as the word2vec, or even classic methods like the “bag-of-words”, or term-frequency inverse document frequency model (TF-IDF) can be applied. For graph-only models such as the MPSN, a node embedding representation learning model such as DeepWalk or node2vec can be applied Perozzi et al. (2014), Grover and Leskovec (2016). In other cases, such as the hybrid models, it is not clear what the best way to proceed is e.g., one could independently embed the text and graph components and concatenate the feature vectors, or train some kind of joint model. We detail these possibilities next, with more details on “hybrid” models and results in Section 5.

First, concerning the text-centric models, two such models can be considered. In the first model, an event *E* was simply represented by its description or “summary” field. In the second model, arbitrary string field values (which have non-numeric primitive types, thereby precluding the use of dates and integers that may cause noise in such representations) can be “concatenated” together to form a single text field. The idea is to supplement the description where it is sparse, or not distinctive enough between events. For either model, the final “value” for the event is a single text string, and the event database is exactly like a document corpus in an NLP setting.

Representation learning on such a corpus at the document level can be achieved in two ways, one adaptive and the other non-adaptive. The non-adaptive model is the bag-of-words model (also called tf-idf). It has been used prolifically in information retrieval and document classification settings over the decade, and remains both scalable and interpretable. However, one issue with its usage is that the dimensions of the tf-idf vectors are sparse, large and not able to easily generalize to unseen words, or even words with similar meaning. Statistically, these issues were not problematic when the corpus was extremely large, such as search engines can often avail of.

More recently, however, adaptive models such as word embeddings have achieved scale, while addressing the dimensionality and sparsity issues, and thereby achieving better performance without necessarily requiring more data. Specifically, the adaptive model “learns” representations of individual words using a neural network like skip-gram. A second model is the continuous bag of words (CBOW) model, but it has typically found to be outperformed by skip-gram. For more details on CBOW, and also other representation learning methods, we refer the reader to an earlier paper on word2vec Mikolov et al. (2013). Our text embedding relies on a more recent version of word2vec called fastText or “bag-of-tricks embedding” that was released openly by Facebook AI research and is able to more gracefully handle misspelled and unusual words Joulin et al. (2016).

Concerning representation learning on the MPSN model, we note that it is more complex than the text, but can be embedded using a graph embedding algorithm. Most graph embedding algorithms are technically designed for uni-partite or uni-type graphs, as is often observed for social networks where every entity is (for testing purposes) an individual, and every link is akin to a friendship (or follower/followee) link. While one could (in theory) devise complex schemes from scratch for multi-partite graphs, a consensus on such representation learning models has yet to emerge in the machine learning community. Therefore, for the purposes of the empirical study and methodology in this paper, we “treat” the MPSN as an ordinary, undirected network. In turn, this implies that all nodes (and not just *event_id* nodes in the event partition) will get embedded by the algorithm; however, only *event_id* node embeddings will get used during the ranking phase. For the embedding algorithm itself, we use DeepWalk Perozzi et al. (2014), although algorithms like node2vec Grover and Leskovec (2016) could also be considered by future researchers to obtain potential performance increases.

An important point to note about all methods presented thus far is that they work in an unsupervised fashion i.e., no “training” links need to be observed for the system to predict them during test time. Initially, therefore, no two event_ids in the network are directly linked, though many have indirect links (e.g., if they share a location). The reason that unsupervised link prediction between events is important was mentioned earlier, namely, geopolitical events such as terrorist attacks tend to be special, irregular and “black swan” events by definition Nassim (2007), and it is not practical to assume that a machine (especially, deep) learning system can be trained on a sparse set of events and still be expected to generalize well. Although investigating the issue of transfer learning (as applicable to this task) is a promising direction Zhuang et al. (2020), it is beyond the scope of this current work and we leave it for future research to pursue.

### 3.4 Quality Evaluation Metrics

We consider several metrics that are regularly employed both in the link prediction as well as in the knowledge graph embeddings literature. These metrics include *Hits@10*, *Precision@k*, *Recall@k*, and *Mean Rank*. These metrics are defined more completely below, but a common aspect of the metrics is that they are used to evaluate mechanisms that take an event as input, and output a ranked list of events as output. When events are represented as vectors, cosine similarity is used to generate the rankings. Namely, given an input (event) vector *e*_*in*_, the ranked list *E*_*out*_ = [*e*_1_, …, *e*_|*E*|−1_] is generated^10^ by computing the cosine similarity between *e*_*in*_ and each event vector in *E* − *e*_*in*_, where *E* is the set of all events. The ranked list obeys the rule *cosineSim*(*e*_1_, *e*_*in*_) ≥…≥ *cosineSim*(*e*_|*E*|−1_, *e*_*in*_). However, for some metrics (Hits@10 and Mean Rank) it is standard to ‘filter’ the set *E* for a linked event pair (in the ground truth) (*e*_*in*_, *e*_*i*_) by removing from *E* all events *except* *e*_*i*_ that are *also* linked with *e*_*in*_. This ensures that the ideal rank for *e*_*i*_ (given *e*_*in*_ as input) should always be 1, since there is no danger that another ‘relevant’ entry is above it in the ranked list. We now define the metrics:

*Hits@10*: Given an event *e*_*in*_ as input, and a (with-held) ground-truth linked pair (*e*_*in*_, *e*_*i*_), the Hits@10 metric measures whether *e*_*i*_ is in the top-10. It is important to note that Hits@10 is evaluated independently for each pair of events linked in the ground truth. As we noted in the example above, an event can be linked with more than one event, which necessitates removing true positives (except the true positive that is in the pair) from the full set of events before evaluating the ranking.

*Mean Reciprocal Rank (MRR)*: The MRR is the reciprocal of the rank at which *e*_*i*_ occurs. Unlike Hits@10, it can be non-zero if *e*_*i*_ is not in the top-10 though it declines very quickly. MRR is evaluated in a similar way to Hits@10 in that the event set has to be filtered prior to ranking for a given input event and a withheld linked pair of events. Because of the event filtering, the optimal MRR is always 1.

*Normalized Discounted Cumulative Gain (NDCG)*: The MRR has several issues, the most important of which is that it is designed to work for only one relevant item per input, and declines quickly the further away that relevant item is from the top of the list. The NDCG is widely used in the information retrieval community as a more robust measure. Unlike the previously described metrics, NDCG does not assume that a given event is only linked to one other event, and hence, filtering is unnecessary.

To compute the NDCG, we first have to calculate the DCG for input event *e*_*in*_, defined by the following equation:

$$DCG_{e_{in}}=rel_{1}+\overset{n}{\underset{p=2}{\sum }}\frac{rel_{i}}{\log_{2}\left(i+1\right)}$$ (1)

Here, *rel*_*i*_ is the relevance of the *i* ^*th*^ item in a ranked list of size *n*. In our case, this is either a 1 (if the event in *E* − *e*_*in*_ at that rank is paired with *e*_*in*_ in the GTD link prediction ground truth) or a 0. We can compute the DCG of both the actual ranking and of an *ideal* ranking (where all relevant items are ranked at the top), the latter denoted as the IDCG (Ideal DCG). The NDCG is then given by:

$$NDCG_{e_{in}}=\frac{DCG_{e_{in}}}{IDCG_{e_{in}}}$$ (2)

Note that the NDCG is between 0.0 and 1.0, since the DCG is always less than the IDCG. Similar to MRR and Hits@10, to obtain performance over the entire set of input events in the ground-truth, we average the NDCG obtained per input event.

We note finally that for each of the models described in the previous section, the evaluation is on a uniform footing because 1) each method is *unsupervised*; 2) each method *represents* an event as a vector^11^; 3) the ranked list (for an input event) for each method is generated in an identical way, namely using cosine similarity. In turn, this implies that, within the scope of the event link prediction task, we can use the results to evaluate the power of the representation (and where applicable, its ‘learning’ using contexts and neural networks) itself.

## 4 Results

Table 3 reports results for the text-centric models introduced in Section 3.2. We consider using only the “summary” or description field, as discussed therein, as well as the concatenation of all text-based fields, which includes both the summary, as well as the “location” field. Note that other fields, such as attack type, date, and so on, are categorical or numerical. Two important things stand out from the table. First, text-based methods generally do quite well, as long as the summary is included. As might be expected, using location alone leads to very noisy results^12^. Second, we find that the classic tf-idf method is difficult to outperform, with the embedding-based method doing significantly worse no matter the experimental setting. In other work, the embedding-based method usually outperforms the tf-idf, although the margin depends significantly on the dataset. It is possible that transformer-based models such as BERT may end up outperforming the tf-idf but we leave an evaluation of this hypothesis for future research. Overall, the results are quite promising: an MRR of 57.88% (the best result, using the simplest possible combination of tf-idf on the summary field) implies that, on average, given an input event, the best method is able to retrieve a relevant result between ranks 1 and 2. The NDCG suggests that the performance gets even better once we consider the unfiltered version of the dataset wherein an input event can have multiple relevant events linked to it in the ground-truth.

**TABLE 3:** Results of text-rich systems on the event link prediction task. Metrics are described in Section 3.4. In all cases below, the ranking is generated using the cosine similarity function between the vectors.

Representation method	Field(s) being represented	MRR	Hits@10	NDCG
tf-idf	Summary	0.5788	0.9821	0.7482
Bag-of-tricks embeddings	Summary	0.5247	0.9043	0.6883
tf-idf	Summary + Location	0.5593	0.9657	0.7339
Bag-of-tricks embeddings	Summary + Location	0.5149	0.8944	0.6838
tf-idf	Location	0.0406	0.0659	0.1111
Bag-of-tricks embeddings	Location	0.0377	0.05995	0.1017

Table 4 reports results for the MPSN methods. We find that performance is significantly worse than the text-based methods; however, as more information is included in the MPSN model, the performance starts increasing. This suggests that the problem is one of information scarcity, not faults with the model or representation learning itself. It also provides some guidance on the ‘information gap’ between the structured attributes, such as attack type (AT) and target type (TT) compared to the text. Indeed, in comparing the *AT + TT + Country + Region* results to the next two information sets in the table, we find the critical importance of the temporal component of the event. Unfortunately, many NLP algorithms that extract events from text focus more on actors and triggers rather than on temporal prediction. For such extractions to have value in graph-based link prediction tasks, more attention needs to be given to accurately populating spatiotemporal spans of events, and on acquiring sufficiently robust descriptions, perhaps by applying a summarization algorithm on event field reports Nenkova and McKeown (2012).

**TABLE 4:** Results of graph-based methods on the event link prediction task. The representation learning in all cases was the DeepWalk algorithm.

Layers used	MRR	Hits@10	NDCG
Attack Type (AT)+Target Type (TT)	0.0054	0.0112	0.0575
AT + TT + Country + Region	0.0474	0.1375	0.2032
AT + TT + Date	0.2936	0.7428	0.5342
AT + TT + Country + Region + Date	0.3678	0.8637	0.6278

## 5 Discussion

Results in the previous section showed that there is obvious value in both structured attributes, and in the text descriptions, although the latter seems to contain more information than the former for machine learning purposes. In Section 3.2, we suggested the possibility of “combining” the text-centric model with an MPSN in what was referred to as a “hybrid” model. Herein, we briefly illustrate two separate results, one that uses a *joint* model and another that uses an *ensemble* model.

Each of these two models accepts as input one of the structured information sets in Table 4 and the ‘Summary’ field. The ensemble model concatenates the bag-of-tricks embedding (which is set to have the same dimensionality as the DeepWalk network embedding applied on the MPSN) with the MPSN network embedding. We then apply the cosine similarity, as earlier, except that the vector is a concatenation of two vectors^13^. The joint model, in contrast, adds the summary field as another “information set” layer in the MPSN model illustrated in Figure 3. Because the field is text-based, rather than structured, we combine its sentences with the random walks output by DeepWalk prior to the algorithm applying the classic word2vec on the random walks. The joint model is therefore hierarchical: it combines the sentences in the summary field with the random walks, thereby embedding words and vertices in a joint setting. The results for both models are illustrated in Table 5.

**TABLE 5:** Results of hybrid methods on the event link prediction task. E and J respectively indicate whether the method is an “ensemble” or “joint” method.

Graph method	E	J	MRR	Hits@10	NDCG
AT + TT	—	X	0.1864	0.3761	0.2366
AT + TT + Country + Region	—	X	0.2077	0.4412	0.3294
AT + TT + Date	—	X	0.3184	0.7341	0.5530
AT + TT + Country + Region + Date	—	X	0.3772	0.8467	0.6282
AT + TT	X	—	0.0901	0.2244	0.1717
AT + TT + Country + Region	X	—	0.1221	0.3262	0.2827
AT + TT + Date	X	—	0.3786	0.8343	0.6156
AT + TT + Country + Region + Date	X	—	0.4168	0.9238	0.6623

We find again (analogous to the results in Table 4) that the best results are achieved when the full information set is used, with the ensemble model achieving an almost 4% improvement on NDCG and MRR compared to the joint model. While both text and graph attributes have value, combining them in a single embedding framework clearly requires more thought, and an independent summing (as the ensemble model achieves) may be a safer approach in the absence of a large training dataset that could be used to fine-tune such a model. We also find that none of the methods, even in the hybrid setting, outperforms the classic tf-idf using just the summary field, illustrating that, on difficult problems like geopolitical event link prediction that are sufficiently different from benchmark link prediction problems often encountered in the literature, the utility of classic methods cannot be discounted. However, there is still much work to be done on the performance front since no method exceeds an NDCG of 80%. Another promising line of future work is to consider a *supervised* version of the problem wherein, in lieu of using cosine similarity on embeddings in an unsupervised framework, a classifier would be trained using a limited quantity of provided linked events (training data), with the embeddings as *features*. Such a classifier should yield better performance than the unsupervised methods presented in this article as initial approaches. Within the supervised learning paradigm, metrics such as accuracy, precision, recall and F1-Measure could also be applied to assess linking quality.

## 6 Conclusion

In this paper, we introduced and presented an empirical study on the problem of event link prediction. We presented various viable models for addressing the problem, derived from established literature on representation learning, followed by a detailed set of results using metrics inspired by the information retrieval community that has previously been applied mostly to Web retrieval and social networks.

Many questions still remain and constitute valuable opportunities for future research. First, it is not clear if the superior performance of text representations (even using simple bag-of-words approach) is fundamentally because the text contains much more information than the graph attributes do or because we have not designed or applied a sufficiently powerful representational model. For instance, it may very well be the case that the particular multi-partite semantic network model that we considered for representing an event-record is unsuitable, and a different model may prove to be more suitable. Similar concerns may apply to the representation learning algorithm used. Teasing apart these various effects is an empirical exercise. Theoretically, much work remains to be done on understanding how various algorithms and models in the machine learning and NLP communities apply differently to events rather than to entities.

## Data Availability

The original contributions presented in the study are included in the article/Supplementary Material, further inquiries can be directed to the corresponding author.

## Footnotes

1 Two examples being Nate Silver’s FiveThirtyEight, and the New York Times’s forecasting interface.

2
https://acleddata.com/

3
https://dss.princeton.edu/catalog/resource1507

4
http://eventdata.parusanalytics.com/data.dir/cameo.html

5 Although in this article, we primarily focus on link prediction as the primary use case.

6 In much of the NLP work, this statement is obvious enough that it is never stated explicitly: e.g., word2vec assumes that the document corpus is a large set of word ‘sequences’, with the skip-gram model used for representation learning, while the ‘bag-of-words’ approach models each document as a multi-set of words, with vector weights derived using the famous tf-idf formula.

7
http://www.start.umd.edu/gtd/

8 Events for which at one link was available.

9 Even more recently, so-called *transformer-based* neural networks such as BERT and GPT-3 Devlin et al. (2018), Brown et al. (2020) have also been used with great effect in NLP tasks. Their application to graph applications and event link prediction remains untested, and they are expensive to fine-tune (requiring supervision). We leave their exploration for this problem domain for future research.

10 The index of the last event in the ranked list if |*E*| − 1 because the input event is not included in the ranked list.

11 This is also true of the tf-idf model, even though the vector is not derived using a neural network, and for that reason (in keeping with terminology as it is commonly used today), is not considered as an ‘embedding’.

12 However, there is some signal in location clearly, since even a performance of 4% on MRR, as the tf-idf achieves using location alone is far better than what would be achieved through a random ranking of events in *E* − *e*_*i*_ *n* per input event *e*_*i*_ *n*.

13 Mathematically, this is equivalent to summing the cosine similarities obtained independently from the text embedding and graph embedding components; hence, the model is referred to as an ensemble model.
